# Use of High-Flow Nasal Cannula Oxygen Therapy in a Pregnant Woman with Dermatomyositis-Related Interstitial Pneumonia

**DOI:** 10.1155/2017/4527597

**Published:** 2017-12-31

**Authors:** Tomohiro Shoji, Takeshi Umegaki, Kota Nishimoto, Natsuki Anada, Akiko Ando, Takeo Uba, Munenori Kusunoki, Kanako Oku, Takahiko Kamibayashi

**Affiliations:** Department of Anesthesiology, Kansai Medical University Hospital, Osaka, Japan

## Abstract

A 33-year-old pregnant woman was referred to our hospital with respiratory distress at 30 weeks of gestation. Chest computed tomography (CT) scans revealed pulmonary infiltrates along the bronchovascular bundles and ground-glass opacities in both lungs. Despite immediate treatment with steroid pulse therapy for suspected interstitial pneumonia, the patient's condition worsened. Respiratory distress was slightly alleviated after the initiation of high-flow nasal cannula (HFNC) oxygen therapy (40 L/min, FiO_2_ 40%). We suspected clinically amyopathic dermatomyositis (CADM) complicating rapidly progressive refractory interstitial pneumonia. In order to save the life of the patient, the use of combination therapy with immunosuppressants was necessary. The patient underwent emergency cesarean section and was immediately treated with immunosuppressants while continuing HFNC oxygen therapy. The neonate was treated in the neonatal intensive care unit. The patient's condition improved after 7 days of hospitalization; by this time, she was positive for myositis-specific autoantibodies and was diagnosed with interstitial pneumonia preceding dermatomyositis. This condition can be potentially fatal within a few months of onset and therefore requires early combination immunosuppressive therapy. This case demonstrates the usefulness of HFNC oxygen therapy for respiratory management as it negates the need for intubation and allows for various treatments to be quickly performed.

## 1. Introduction

High-flow nasal cannula (HFNC) oxygen therapy is widely used in the management of acute respiratory failure and also has applications in cases with acute exacerbation of interstitial pneumonia (IP) [1–3]. Although pregnant patients with IP rarely develop concurrent complications of polymyositis (PM) or dermatomyositis (DM), the prompt diagnosis of PM/DM in these patients is critical due to the high risk of potentially fatal outcomes to both the mother and the fetus [4–6]. This case report describes the use of HFNC oxygen therapy without intubation in a 33-year-old pregnant woman who developed progressive IP complicated by DM at 28 weeks of gestation. The patient was successfully treated with combination immunosuppressive therapy.

## 2. Case Report

A 33-year-old pregnant woman was admitted to our hospital due to respiratory distress at 30 weeks of gestation. The patient had previously undergone three vaginal deliveries. A review of family history revealed that the patient's paternal grandmother had rheumatoid arthritis and the patient's father had unspecified IP. The patient first experienced respiratory distress in her 28th week of gestation; her condition deteriorated two weeks later, and she was transported to our hospital via ambulance. Upon admission, the patient was lucid and afebrile (36.5°C). The respiratory and hemodynamic levels are revealed in Table 1. She had blood pressure of 88/49 mmHg, a heart rate of 86 bpm, a respiratory rate of 18 breaths/minute, and peripheral oxygen saturation (SpO_2_) on room air of 90%. Fine crackles were noted in both lower lung fields.

Laboratory examination revealed slight elevations in white blood cell count (11,900/*μ*l), serum C-reactive protein concentration (2.65 mg/dl), and aldolase level (7.1 U/l). Serum KL-6 level was highly elevated at 986 U/ml. An arterial blood gas test showed poor oxygenation with arterial oxygen partial pressure (PaO_2_) on room air of 61.7 mmHg.

**Figure 1:**
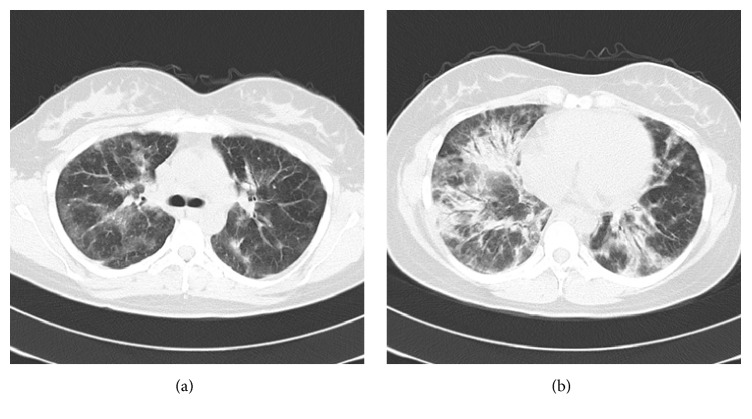
Chest CT scans showing the patient's middle (a) and lower (b) lung fields upon admission. Bilateral pulmonary infiltrates along the peripheral bronchovascular bundles and ground-glass opacities with a panlobular distribution were observed.

Although the patient had eczema and ulceration on the dorsal surface of both hands on the first day of hospitalization, she did not present with Gottron's sign or muscle weakness, which are characteristic of DM. Chest computed tomography (CT) scans (Figure 1) revealed pulmonary infiltrates along the bronchovascular bundles and panlobular ground-glass opacities in both lungs. N-terminal (NT) pro-B-type natriuretic peptide (BNP) level was at 258.8 pg/ml without renal dysfunction. Cardiac dysfunction was not revealed except for slight dilatation of the left ventricle. The differential diagnosis included idiopathic IP and IP complicated by a collagen disease such as DM. Due to the rapid progression of respiratory distress within a short period of time, the patient was given intravenous methylprednisolone pulse therapy (1 g/day) from the first day of hospitalization. Despite this treatment, the patient's condition worsened on the second day of hospitalization, and she developed orthopnea (grade V based on the Hugh-Jones classification). As a result, HFNC oxygen therapy was initiated at 30 L/min with a fraction of inspired oxygen (FiO_2_) of 0.30 according to the instructions of the intensivists and anesthesiologists. However, this did not improve respiratory distress with SpO_2_ remaining at 90%. HFNC oxygen parameters were increased to 40 L/min with FiO_2_ at 0.40, and respiratory distress began to improve (SpO_2_: 92–94%).

Due to the rapid disease progression and resistance to steroid treatment, we suspected IP complicated by PM/DM or clinically amyopathic DM (CADM), which is a form of DM without overt signs of myositis. Accordingly, we deemed it necessary to begin immunosuppressive therapy. At 30 weeks of gestation, the fetal body weight was over 1500 g, and it was determined that the neonate could be treated at the neonatal intensive care unit after delivery. HFNC oxygen therapy was continued, and an emergency cesarean section without use of tocolytics was performed under spinal anesthesia. The neonate weighed 1550 g, and the Apgar scores at one minute and five minutes after birth were 8 and 9, respectively. Tracheal intubation was not required during the procedure. A chest X-ray indicated that the pulmonary infiltrates had spread further. Due to this exacerbation of IP, we began treatment with ciclosporin (0.2 g/day) from the third day of hospitalization. On the following day, we observed newly formed heliotrope rash on both upper eyelids and keratotic rash along the surface of the fingers of both hands. Chest CT scans confirmed that the infiltrates had expanded since the patient was admitted, and cyclophosphamide pulse therapy (1 g/month) was added to the patient's regimen on the fifth day of hospitalization.

**Figure 2:**
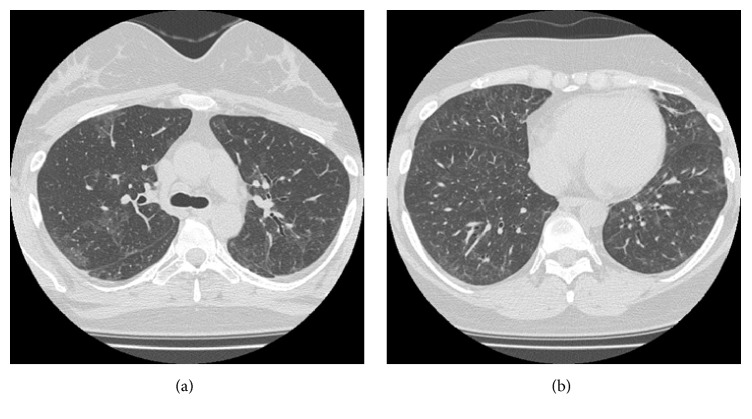
Chest CT scans showing the patient's middle (a) and lower (b) lung fields after two months from the initiation of treatment. The pulmonary infiltrates had disappeared.

With HFNC oxygen therapy (40 L/min, FiO_2_: 0.40), respiratory distress was alleviated (SpO_2_: 93–96%). Three days after the cesarean section (fifth day of hospitalization), the patient was transferred from the intensive care unit to a general ward. On the seventh day of hospitalization, the results of analysis of a blood sample taken on the day of admission showed that the patient had positive titers for autoantibodies against aminoacyl tRNA synthetase (ARS), including anti-Jo-1 antibodies. Due to the presence of anti-ARS antibodies, we excluded the possibility of CADM. The patient's respiratory condition further improved, and the parameters of HFNC oxygen therapy were reduced to 30 L/min with FiO_2_ at 0.30. A chest CT scan taken after 18 days of hospitalization indicated a new case of pneumomediastinum, but the interstitial shadows in the lung field had substantially receded. The patient was discharged after 47 days of hospitalization. Following 2 months of treatment, chest CT scans showed that the pneumomediastinum and the interstitial shadows had disappeared (Figure 2). During that time, the patient complained of polyarthralgia. Together with the other symptoms of heliotrope rash, elevated aldolase level, elevated C-reactive protein concentration, and positive titers for anti-ARS antibodies, the inclusion of arthralgia fulfilled the diagnostic criteria for DM as stipulated by Japan's Ministry of Health, Labour and Welfare based on Tanimoto et al. [7]. The final diagnosis was IP preceding DM with delayed manifestation of specific cutaneous findings without overt signs of myositis.

## 3. Discussion

This case provided valuable findings that the use of HFNC oxygen therapy was able to contribute to the alleviation of respiratory distress in rapidly progressive IP. To the best of our knowledge, this report describes the first case of rapidly progressive IP where hypoxia was successfully prevented in both the mother and the fetus without intubation.

It has been reported that the use of HFNC oxygen therapy in acute respiratory failure cases did not result in lower intubation rates relative to oxygen therapy delivered through a face mask and noninvasive positive-pressure ventilation, but it was associated with more ventilator-free days and a higher survival rate [3]. In addition, HFNC oxygen therapy allows for patients to eat, drink, and move around without the need to interrupt treatment [8]. Our case did not require intubation throughout the cesarean section procedure and immunosuppressive therapy, which allowed her to have meals, converse with others, and interact with her child. In addition to alleviating respiratory distress, the use of HFNC oxygen therapy may have reduced the patient's feelings of anxiety and improved her quality of life during hospitalization. This form of respiratory management should therefore be considered for other similar cases in the future.

The association between the prognosis of IP patients with DM and the degree of myositis disease activity has been previously documented, and the early use of immunosuppressants should be employed in cases with rapidly progressive IP [5]. When refractory IP is complicated by PM/DM or CADM, the pulmonary tissue may become irreversibly damaged. As a result, the condition may become resistant to combination immunosuppressive therapy and eventually lead to death after only several months [5, 9, 10]. As our patient's respiratory condition continued to worsen despite immediate steroid pulse therapy, we suspected refractory IP complicated by PM/DM or CADM, and the early use of immunosuppressive therapy was deemed necessary. While the diagnosis of DM was made later, the possibility of refractory IP preceding PM/DM or CADM prompted us to consider the early use of combination immunosuppressive therapy.

It should be noted that the use of immunosuppressants for the treatment of IP does not ensure rapid improvement in patient condition. In a similar case report, a pregnant woman at 16 weeks of gestation had developed IP preceding PM and was treated with a combination of steroid pulse therapy and tacrolimus [11]. Due to that patient's worsening respiratory condition, the pregnancy was terminated in the 21st week of gestation to save the mother. Cyclophosphamide pulse therapy was subsequently added to the treatment regimen, and the patient began to show signs of improvement. Cases of IP complicated by PM/DM or CADM in pregnant women are extremely rare. Therefore, it remains unclear if the use of combination immunosuppressive therapy (including cyclophosphamide) would produce quick therapeutic effects in cases without termination of pregnancy.

Cardiac involvement has been reported in patients with DM, and the incidence has reached as high as 45.7% [12]. Moreover, interstitial pneumonia has been reported as one of the major predictive factors of cardiac dysfunction in patients with DM [12]. Left ventricular diastolic dysfunction is an early feature of cardiac involvement in patients with PM/DM [13], and cardiac involvement is a common cause of death [14]. This case has not clinically revealed cardiac dysfunction, but diastolic dysfunction might have potentially progressed because of elevation of NT-pro BNP, slight dilatation of the left ventricle, and alveolar syndrome with air bronchogram on the CT chest. HFNC might have suitably applied positive end expiratory pressure [15]. HFNC oxygen therapy was seamlessly provided without interruption throughout the patient's treatment in the intensive care unit, the operating theater, and the general ward and during transfers between these units. As the pregnancy had progressed to the point where the baby could be treated in the neonatal intensive care unit after delivery, the nonuse of intubation allowed the patient to be quickly transitioned from the cesarean section to immunosuppressive therapy. This way, we were able to save both the mother and the child.

## 4. Conclusions

This case report describes the successful use of HFNC oxygen therapy for respiratory management in a pregnant patient who developed rapidly progressive IP complicated by DM. Both the mother and the child were saved. It is necessary to quickly treat such cases with a combination of steroid pulse therapy and immunosuppressants. HFNC oxygen therapy is a useful respiratory management method that negates the need for intubation and allows for greater freedom of treatment and patient comfort.

## Figures and Tables

**Table 1:** The respiratory and hemodynamic levels from hospital admission to ICU discharge.

Variables	Oxygen therapy	SpO_2_ (%)	PaO_2_ (mmHg)	Respiratory rate (min^−1^)	Systolic blood pressure (mmHg)
Hospital admission	Room air	90	61.7	18	88
ICU admission	HFNC 40 L/min, FiO2 0.40	94	64.5	28	111
ICU day 2	HFNC 40 L/min, FiO2 0.40	95	73.5	19	94
ICU day 3	HFNC 40 L/min, FiO2 0.40	95	73.3	17	124
ICU day 4	HFNC 40 L/min, FiO2 0.40	96	89.3	17	122

ICU: intensive care unit; SpO_2_: oxygen saturation of peripheral artery; PaO_2_: partial pressure of arterial oxygen; HFNC: high-flow nasal cannula; FiO2: fraction of inspiratory oxygen.
